# EEG-Based Identification of Emotional Neural State Evoked by Virtual Environment Interaction

**DOI:** 10.3390/ijerph19042158

**Published:** 2022-02-14

**Authors:** Dawoon Jung, Junggu Choi, Jeongjae Kim, Seoyoung Cho, Sanghoon Han

**Affiliations:** 1 Yonsei Graduate Program in Cognitive Science, Yonsei University, Seoul 03722, Korea; dw.jung@yonsei.ac.kr (D.J.); junggu.choi@yonsei.ac.kr (J.C.); jeongjaekim@yonsei.ac.kr (J.K.); seoyoung.cho@yonsei.ac.kr (S.C.); 2 Department of Psychology, Yonsei University, Seoul 03722, Korea

**Keywords:** electroencephalography, virtual reality, emotion recognition, machine learning

## Abstract

Classifying emotional states is critical for brain–computer interfaces and psychology-related domains. In previous studies, researchers have tried to identify emotions using neural data such as electroencephalography (EEG) signals or brain functional magnetic resonance imaging (fMRI). In this study, we propose a machine learning framework for emotion state classification using EEG signals in virtual reality (VR) environments. To arouse emotional neural states in brain signals, we provided three VR stimuli scenarios to 15 participants. Fifty-four features were extracted from the collected EEG signals under each scenario. To find the optimal classification in our research design, three machine learning algorithms (XGBoost classifier, support vector classifier, and logistic regression) were applied. Additionally, various class conditions were used in machine learning classifiers to validate the performance of our framework. To evaluate the classification performance, we utilized five evaluation metrics (precision, recall, f1-score, accuracy, and AUROC). Among the three classifiers, the XGBoost classifiers showed the best performance under all experimental conditions. Furthermore, the usability of features, including differential asymmetry and frequency band pass categories, were checked from the feature importance of XGBoost classifiers. We expect that our framework can be applied widely not only to psychological research but also to mental health-related issues.

## 1. Introduction

The identification of neural states plays an important role in a variety of domains including brain–computer interaction (BCI) and psychological research [1,2,3]. In several previous studies, diverse methodologies have been applied to measure neural state variations among participants. Kevric and Subasi [4] proposed a motor imagery BCI model using electroencephalography (EEG) signals. Giuseppina et al. [5] utilized brain functional magnetic resonance imaging (fMRI) data to construct a BCI system. Among several brain imaging techniques used in associated research, the EEG-based approach is primarily used to analyze neural activities. In Xiaotong et al. [6], the authors suggested high temporal resolution reaching the millisecond level as advantages of EEG signals in associated research. Additionally, the signal reliability and mobility of recently used EEG collection devices have been positively evaluated for real-world BCI and clinical usage.

Based on the aforementioned advantages of EEG signals, several researchers collected EEG signals and used them to compare or classify the psychological states of participants. Duan et al. [7] attempted to classify the emotional states of participants using EEG signals. Fluctuations in EEG signals were recorded with movie clips used as stimuli. Secerbegovic et al. [8] used a single-channel EEG signal to differentiate between mental workload and stress in student groups. To investigate the different neural state variations in stress and workload, two scenarios were provided for distinct groups. The student group conducted 3-back visual tasks with high difficulty in the first scenario. For the other group, the same visual tasks (i.e., 3-back visual task) and Lumosity games were offered sequentially. Dong et al. [9] evaluated the spatial cognitive ability based on EEG signal analysis. They recorded and compared EEG signals before and after a spatial navigation training program. Navigation tasks for visible/hidden routes in a virtual city street environment were used for evaluation.

In recent research, virtual reality (VR) has been widely used as an experimental or therapeutic material. Kim et al. [10] proposed a human perception test framework for Hemispatial neglect based on VR. The test framework consists of screens with targets in 3D environments. Selections of participants about the target were recorded to assess their perception abilities. Elizabeth et al. [11] utilized VR tools to investigate the efficacy of exposure therapy for posttraumatic stress disorder (PTSD). Exposure therapy with imaginal exposure was offered in the display of VR devices. To compare the level of PTSD, clinician interviews were conducted before and after treatment. Torrico et al. [12] examined the emotional responses to chocolate in VR environments. Three environments, including traditional booths and VR devices, were used to identify differences in the effects of sensory responses.

Various virtual environments with EEG signals have been considered as research methods in the associated research. To check neural state variations in VR conditions, the measured EEG signals were analyzed by reaction from participants. Yu et al. [13] organized research designs using VR emotion recognition tasks and EEG measurements. Three different categories of stimuli (positive, neutral, and negative) were included in the tasks to compare emotional responses in experimental conditions. Functional brain network indices were calculated to detect the influence of tasks in a virtual environment. Tamburin et al. [14] used VR settings in their research to check the associations between craving and EEG measurements. Among diverse craving situations, the authors evaluated smoking-related cue reactivities in smokers and non-smokers. The participant groups (i.e., smokers and non-smokers) were exposed to two VR scenarios (mountain landscape and office). Band-pass power values were processed from EEG signals collected in virtual scenarios to distinguish the effects of the cues.

Li and Kim [15] evaluated the effects of task complexity in a VR environment using EEG signals. They calculated descriptive statistics from the behavioral responses. To compare responses between conditions, the authors conducted a repeated-measures ANOVA test. Kisker et al. [16] gathered electrophysiological correlates of fear and avoidance tendencies from EEG signals. Subsequently, Shapiro–Wilk tests were used to check the normality of the variables. Moreover, ANOVA and t-tests were applied to investigate the differences between responses and EEG analysis results.

Various studies have applied machine or deep learning algorithms to find latent patterns in EEG data. Qureshi et al. [17] utilized K-nearest neighbor (KNN) and fuzzy rough nearest neighbor (FRNN) algorithms to classify epileptic seizures from EEG signals. They suggested EEG feature extraction algorithms based on discrete wavelet transformation (DWT) methods. Additionally, the classification performances of fuzzy function-based algorithms were compared with traditional algorithms (such as decision tree and random forest). Furthermore, Geraedts et al. [18] proposed a fully automated EEG-based machine-learning pipeline to identify patients with PD. The proposed pipeline showed a better classification performance than the other frameworks. 

Based on previous studies, we proposed a machine learning framework for neural state classification in a virtual environment based on EEG analysis. EEG signals were collected from 15 participants before and after the tasks to investigate the influences of VR tasks. A total of three virtual environments (high, low arousal, and social anxiety) were provided to participants for the evaluation of emotional responses. To identify optimal classification algorithms for our framework, three machine learning models (k-nearest neighbors, support vector machine, and random forest) were applied to the experiments in our study. Furthermore, we checked the important features of the classification models to interpret our experimental results.

The main contributions of our research are as follows. First, regarding the external environment during EEG signal collection, we provided various virtual situations in VR devices to investigate the influence of virtual content on neural state variation. Second, for checking suitable classification algorithms in our research scheme, we found the appropriateness of the XGBoost classifier based on comparisons between three machine learning classifiers. Third, as regards identification of important features in classification tasks, we verified the usability of fourteen features for classification among fifty-four features widely used in previous studies. Finally, we proposed a machine learning-based emotion classification framework with EEG signals in a virtual environment. This framework can contribute to future research in the field of mental states-related health issues (such as PTSD, obsessive-compulsive disorder, autism spectrum disorder, and attention deficit hyperactivity disorder).

## 2. Materials and Methods

### 2.1. Overview

**Figure 1:**
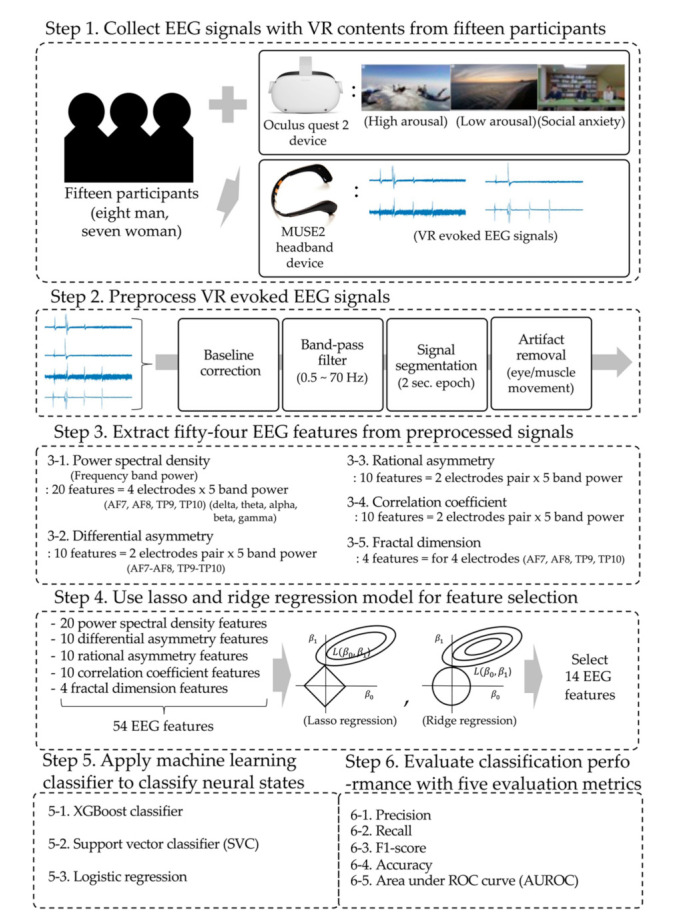
Overview of the research scheme.

Our experimental design comprised six steps. First, we collected VR-evoked EEG signals from participants in three virtual environment conditions. Second, EEG signals were preprocessed to perform feature extraction. Third, 54 features were extracted from the preprocessed EEG signals. Fourth, lasso and ridge regression models were used to select the proper features from the 54 extracted features. Fifth, we applied three machine learning classifiers to classify emotional neural states based on EEG signal features. Finally, the classification performance was evaluated using five evaluation indices. The detailed steps are shown in Figure 1.

### 2.2. Participants

Fifteen participants (eight men and seven women; age range, 19–38 years; mean = 26.27, standard deviation = 5.38) participated in the study. None of the participants reported any mental illness. The experiments were designed and conducted in accordance with the institutional review board approval at Yonsei University. In the experiment, participants were required to experience three types of 360-degree VR content on YouTube to induce different neural states [19,20,21]. Oculus Quest 2, a VR head-mounted display, was used to experience the VR content.

### 2.3. EEG Data Acquisition and Experimental Procedure

The three types of VR content—low arousal, high arousal, and social anxiety—were provided as stimuli. In the low-arousal condition, a virtual calm sea view was shown to experience a low-arousal state. In contrast, environments with skydiving were shown to experience a high-arousal state in high-arousal conditions. A previous study validated that skydiving experiences elicited a hyperarousal state [22]. To arouse a relatively higher emotional state than the aforementioned two conditions, we conducted group interviews under social anxiety conditions. Evoked social anxiety through VR content has been revealed in previous studies [23,24]. 

**Figure 2:**
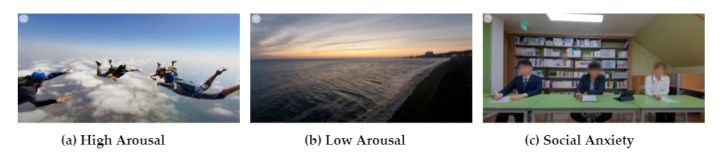
VR content to induce the state.

Participants were instructed to look around their surroundings in high- and low-arousal conditions. In social anxiety conditions, interviews with three interviewers were conducted. The level of arousal could be similar in conditions of high arousal and social anxiety. However, high arousal and social anxiety were distinct in that the condition of social anxiety involved interactions with the interviewer. In addition, the two conditions differed in valence of emotions. Skydiving is a positive experience associated with fun, happiness, and pleasure [25]. The social anxiety condition can induce more negative feelings than the high-arousal condition, since the interview can evoke anxiety and distress on account of the fact that the participants are evaluated by the interviewer [26]. Examples of virtual content are shown in Figure 2.

**Figure 3:**
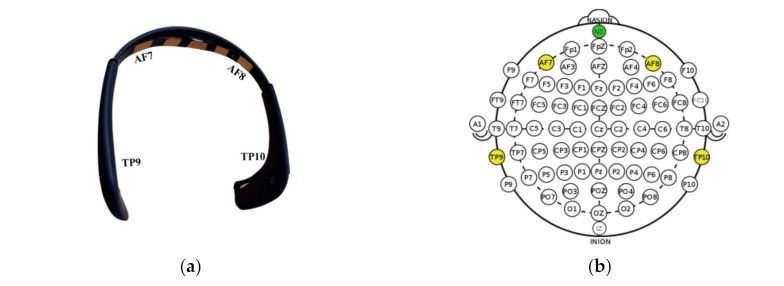
Electrode and channel information of EEG devices used in this study. (**a**) Muse2 headset band. (**b**) EEG montage of Muse 2 headset band based on the International 10–20 EEG electrode placement standard [27,28].

The portable EEG device, which is the MUSE2 headset band from InteraXon, Inc., recorded brain activity. It has seven electrodes: four active electrodes located at AF7, AF8, TP9, and TP10, which are included in the prefrontal and temporoparietal lobe regions, a ground electrode located at FPz in the center, and two reference electrodes to the left and right of the ground (Figure 3). EEG data were acquired using a 256 Hz sampling frequency.

**Figure 4:**
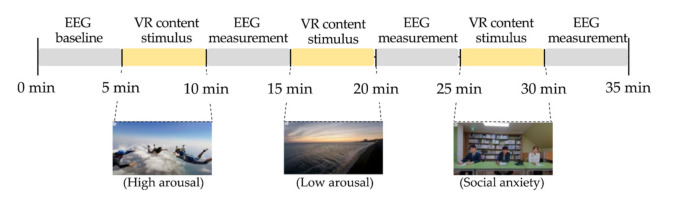
Block diagram of EEG signal collection with VR contents.

Prior to the experiment, we explained the EEG signal collection procedure to the participants and asked each participant to sign a consent form. Once the consent form was signed, eyes-open resting state EEG was recorded for 5 min while the participant sat in a relaxed position to obtain baseline activity. Following the baseline EEG recording, the emotional states following each VR experience were examined for each participant. A block diagram of the collection procedure is presented in Figure 4.

### 2.4. EEG Preprocessing

The VR-evoked EEG signals from the participants were preprocessed using the EEGLAB (interactive MATLAB toolbox) [29]. First, baseline correction was accomplished by subtracting the mean baseline value from each epoch to reduce baseline differences across the EEG channels and temporal drifts. Second, the band-pass filter was applied from 0.5 to 70 Hz in order to minimize artifacts such as direct current shifts and filtering artifacts at epoch boundaries [30]. Third, the continuous resting state EEG data were segmented into a 2 s epoch, the length of which is advisable in order to remove short artifacts and improve non-stationarity [31,32]. Finally, 2 s lengths of epochs with noise or artifacts such as eye movements, eye blinks, and muscle activity were removed manually based on their signal shape (e.g, sudden spikes and similar patterns with cardiac cycles). In addition, an independent component analysis (ICA) algorithm, which identifies maximal temporally independent EEG signals, was used to remove deliberate noise in the EEG signals. As a result, an average of 54.27 epochs remained and was applied for analysis from 150 epoch sets after removal. 

### 2.5. EEG Feature Extraction

We extracted a total of fifty-four features for the analysis. The power spectral density was calculated by averaging the power for the frequency band in each epoch and then averaging it for all epochs. The frequency bands were divided into delta (0.5–4 Hz), theta (4–8 Hz), alpha (8–12 Hz), beta (12–30 Hz), and gamma (30–50 Hz). Twenty frequency power features, that is, five frequency bands for the four electrodes, were extracted. Previous findings have suggested that frequency power analysis of resting-state EEG provides insight into the neural correlates of emotional mental state. For example, frontal delta power is presented during reduced alertness, such as at rest [33]. Lower delta activity in the posterior temporal and occipital areas before target onset is related to a more alert state [34]. Resting state theta activity has been linked to anxiety states, including social anxiety [35,36]. Alpha power is an index of the resting state with reduced cortical arousal. Lower alpha power in the posterior region has been reported in the high-arousal group [37], and high beta power is related to stress and anxiety [38]. Resting state gamma power is associated with high-arousal states, such as higher cognitive and emotional processes [39]. Resting frontal gamma power is associated with early cognition and language [40]. Differential asymmetry (DASM) is the difference in the frequency band power of symmetrical electrode pairs (TP9&TP10 and AF7&AF8), and there are ten DASMs comprising five frequency band powers for the two electrode pairs used [41]. Rational asymmetry (RASM) is the ratio of the frequency band power of symmetrical electrode pairs (TP9&TP10 and AF7&AF8) [42]. A total of ten RASM features, comprising five frequency band powers for two electrode pairs, were used. Correlation coefficients of frequency band power on pairs of electrodes, TP9&TP10 and AF7&AF8, were also used [43]. Thus, a total of ten correlation coefficient features, comprising five frequency bands for two electrode pairs, were used. The fractal dimension (FD) is a measure of the complexity of a key feature of fractals. The nonlinear property of the EEG signal in the time domain can be analyzed using the FD. A low FD corresponds to a regular signal; conversely, a high FD corresponds to an irregular signal. The FD could distinguish the EEG data of subjects in different states [44,45]. The Katz FD was used in this study based on previous results that showed superior performance in the classification of EEG data [46]. The FDs of the four electrodes are calculated in this study.

In summary, the EEG frequency power analysis was performed using a fast Fourier transform. Frequency bands were divided into delta (0–4 Hz), theta (4–7 Hz), alpha (8–12 Hz), beta (12–30 Hz), and gamma (30–50 Hz). The absolute power of each frequency band was estimated for each EEG channel. A total of 20 values (four EEG channels × five frequency bands) was estimated. DASM and rational asymmetry (RASM) are the differences and ratios of symmetrical electrode pairs, which are TP10-TP9 and AF8-AF7. Each pair from five frequency bands (two pairs of EEG channels × five frequency bands) was measured in the DASM and RASM. The correlation coefficient between paired regions was calculated for the five frequency domains (two pairs of EEG channels × five frequency bands). The fractal dimension, which measures the fractal complexity, was calculated for each electrode (four channels). The 54 features mentioned above were analyzed in this study. Detailed extracted features were listed in Table 1.

### 2.6. Feature Selection

Fifty-four features extracted from EEG signals may have two problems. First, a high correlation can be observed between features included in the same categories (multicollinearity or redundancy for classification). Second, irrelevant features for classification can be included in the features (low correlation). To identify the proper features for classification tasks, we applied lasso and ridge regression for feature selection [47,48]. 

In this step, we fitted the lasso and ridge regression models based on a dataset consisting of EEG feature values. Fifty-four features were applied as independent variables and emotional states were used as dependent variables for regression models. The coefficients were checked for individual features and sorted by scale. After sorting features by their coefficients, we selected and compared the top 20 features between the lasso and ridge regression conditions. As a result, 14 common features were selected from 54 features. The selected features are listed in Table 2. 

### 2.7. Machine Learning Classification Algorithm

To classify emotional neural states based on EEG signals, we utilized three machine learning classifiers with 14 EEG features. The first classifier was the XGBoost classifier, which is based on an ensemble of multiple decision tree models. A given dataset with n samples and m features ($D=\{\left(x_{i},\;y_{j}\right)\;|\;x_{i}\in R^{m},y_{j}\in R\}$) was used to train the classifier. In our study, we applied a dataset of 60 rows and 14 features. Gradient boosting methods with regularized objective functions constitute the basis for the algorithm.

(1) $$L\left(\phi \right)=\underset{i}{\sum }l\left(y_{i},\;y_{i}’\right)+\underset{k}{\sum }\Omega \left(f_{k}\right)$$

(2) $$\mathrm{where}\;\Omega \left(f\right)=\gamma T+\frac{1}{2}\lambda {\left|\left|\omega \right|\right|}^{2}$$

(3) $$y_{i}’=\phi \left(x_{i}\right)=\overset{K}{\underset{k=1}{\sum }}f_{k}\left(x_{i}\right),\;f_{k}\in F$$

To optimize the XGBoost classifier with a dataset, we minimize the objective function with regularized terms in (1), where $y_{i}’$ denotes the predicted values from decision tree models and $f_{k}$ indicates individual tree models. Differentiable convex loss functions that compare the difference between the predicted value ($y_{i}’$) and target value ($y_{i}$) were included in (1) as function l. Penalization term was added as function Ω in the second term. In this study, we set $y_{i}$ as class labels to which emotional neural state levels were assigned (such as baseline, low, high arousal, and social anxiety level). 

The second classifier was a support vector classifier (SVC) with nonlinear kernels. This classification algorithm classifies the feature space using hyperplanes to separate class labels. We applied a non-linear kernel (radial basis function kernel) to evaluate the performance under more diverse class conditions. In addition, we trained and evaluated the algorithm performance using completely participant-separated datasets to avoid overfitting when non-linear kernels were used.

The third classifier was the logistic regression model (LR). A maximum likelihood estimation method is used to estimate the coefficients of the regression model. Consequently, the classifier calculates the likelihood value $L\left(x\right)$, where $0\;\leq L\left(x\right)\leq 1$. This likelihood value indicates associations between class labels and input vectors. We considered the basic form of the logistic regression model with our EEG features and neural state classes as follows:

(4) $$F\left(z\right)=E\left(\frac{Y}{X}\right)=\frac{1}{1+e^{-\left(\alpha +\sum \beta_{i}X_{i}\right)}}$$

(5) $$\mathrm{where}\;z=\alpha +\beta_{1}X_{1}+\beta_{2}X_{2}+\ldots +\beta_{k}X_{k}$$

where Y indicates the emotional neural state level as a class label. In summary, logistic regression classifiers suggested the probability of categorizing each class under various conditions.

To determine the optimal hyperparameters of the three classification algorithms, we conducted a random search. The detailed hyperparameters are presented in Table 3.

### 2.8. Evaluation Metrics

We evaluated the classification performance of the three classifiers by comparing five evaluation metric values. The true positive (*TP*), true negative (*TN*), false positive (*FP*), and false negative (*FN*) values were calculated from the confusion matrix. The *TP* and *TN* values indicate the ratio of correctly classified samples. In addition, the *FP* and *FN* values indicate incorrectly classified samples. Finally, four metric values were obtained from the aforementioned four values: precision, recall, f1-score, and accuracy. Additionally, to establish the receiver operating characteristic (ROC) curve, the true positive rate (TPR) and false positive rate (FPR) values were calculated. The area under the ROC curve (AUROC) was determined using an ROC curve.

(6) $$Precision=\frac{TP}{TP+FP}$$

(7) $$Recall=\frac{TP}{TP+FN}$$

(8) $$F1-score=2\times \frac{Precision\times Recall}{Precision+Recall}$$

(9) $$Accuracy=\frac{TP+TN}{TP+FP+TN+FN}$$

(10) $$True\;Positive\;Rate=\frac{TP}{TP+FN}$$

(11) $$False\;Positive\;Rate=\frac{FP}{FP+TN}$$

A fivefold cross-validation was used to evaluate machine learning algorithms in robust settings. Trained data for the classification used 80% of the data, while the remaining 20% were used for testing. The classification scores were averaged across the iterations to estimate the performance of the classifier.

### 2.9. Tools

All codes for ML classifiers and data preprocessing were written using Python (version 3.7.1; scikit-learn, version 2.4.1) and R (version 4.0.3) programming languages.

## 3. Results

### 3.1. Classification Performance of Machine Learning Classifiers 

To identify optimal classification algorithms, we utilized three machine learning classifiers (XGBoost, SVC, and LR) for emotional neural state classification. Classification performance was evaluated based on five evaluation metrics (precision, recall, f1-score, accuracy, and AUROC). In the experimental results, the XGBoost classifiers showed the best classification performance under different conditions. We found the same trends in performance in both binary-class and multi-class conditions. The detailed classification performances and ROC curves are listed in Table 4 and Table 5.

### 3.2. Importance of Features for Classification of Emotional Neural States

We compared the important features of machine learning classifiers to find proper feature categories for classification. Among the three classifiers (XGBoost, SVC, and LR) used in the analysis, the feature importance of XGBoost classifiers was selected based on their classification performance. The top five important features were compared in the binary and multi-class conditions. In five feature categories (FP, DASM, RASM, CC, and FD), features of differential asymmetry (DASM), and frequency band power (FP) categories were mainly found in the experimental results. The detailed important features are listed in Table 6 and Table 7. 

## 4. Discussion

In this study, we attempted to identify emotional neural states based on VR-evoked EEG signals using machine learning classification algorithms. A total of 15 participants were involved in our study to collect EEG signals from four virtual environments. To detect the difference in neural variation between virtual stimuli, fifty-four features including five categories, were extracted. In addition, 14 features were selected based on the coefficients of the lasso and ridge regression models. After consisting of datasets with EEG features, three classification algorithms were used to classify the emotional states. The XGBoost classifier showed the best classification performance for our study design. Furthermore, the feature importance of the XGBoost classifiers was compared to validate the usability of the features for classification. Among the five feature categories, the rank of the DASM and FP features was higher than that of the other categories. To suggest reasonable evidence of our research topics (i.e., VR evoked emotional EEG signals, classification through machine learning algorithms), we found associated previous research. First, related to the collection of EEG signals based on VR content, Tauscher et al. [49] compared EEG signal quality between VR content and traditional displays. They concluded that an improvement in signal quality was found with the fixation of VR contents and straps. Tremmel et al. [50] estimated the cognitive workload in an interactive virtual reality environment using EEG signals. The authors evaluated the feasibility of monitoring cognitive workload via EEG signals while performing a classical n-back test in VR devices. Alimardani et al. [51] assessed empathy levels in an effective virtual environment using EEG signals. The relationships between empathy scores and frontal alpha asymmetry were examined in the experimental results. Second, in terms of analysis with machine learning algorithms, Stevens et al. [52] conducted an analysis of the relationship between EEG and distraction and engagement contents based on machine learning models. An artificial neural network (ANN) model was utilized to determine the influence of various contents on EEG signal values. In Gross et al. [53], researchers attempted to diagnose premature Internet addiction using EEG spectra with machine learning algorithms. A random forest classifier was utilized for the classification of addiction. Baumgartl et al. [54] proposed a measurement system for social desirability based on EEG data. They utilized random forest models to classify social desirability levels. Based on the aforementioned previous studies, we concluded that our research topic regarding emotional neural state analysis evoked by VR through machine learning algorithms was reasonable. In addition, we designed our study based on related previous research to compare or validate the experimental results. Wang et al. [55] attempted to find detailed relationships between different emotional states and various EEG features. To stimulate participants’ emotions, researchers provided a set of movie clips, including two target emotional states (i.e., positive and negative emotions). EEG signals were collected from six health participants while watching movie clips. Three analysis methods (power spectrum, wavelet, and nonlinear dynamical analysis) were used to extract the features. With regard to extracted features, feature dimensionality reduction was conducted using principal component analysis, linear discriminant analysis, and correlation-based feature selector. Support vector machine models were applied to binary classification tasks for the two emotions. The performances of the algorithms were compared under various parameter conditions. Bazgir et al. [56] developed an emotion recognition system using EEG signals. EEG signals were measured with consecutive 1 min length music videos from the participants. The discrete wavelet transform method was used to extract the gamma, beta, alpha, and theta frequency band features. A principal component analysis was applied to maintain the same dimensionality of features. Three classification algorithms (support vector machine, k-nearest neighbor, and artificial neural network) were utilized to classify emotional states. Among the various frequency band features, the beta frequency band condition showed the best classification performance. Similar to the previously mentioned studies, the study design of our research comprised similar steps. First, the EEG dataset was collected in a VR content stimulus from 15 participants. Second, fifty-four EEG features were extracted to evaluate the effects of each feature. Third, we applied a lasso and ridge regression model to select the proper features from fifty-four features. Fourth, XGBoost, SVC, and LR models were used to classify the emotional neural variations. Finally, we compared the classification performances using five evaluation metrics. In our experimental procedure, we compared four levels of emotional neural states in the virtual environment. To set a baseline of the emotional state, we measured the EEG signals without any stimulus materials. Additionally, in low- and high-arousal conditions, EEG signals were collected in virtual environmental stimuli without interaction. Furthermore, neural states in social anxiety were investigated with relatively higher emotional conditions than other conditions in virtual interaction materials. To check the optimal algorithms for our research topic, we validated the classification performances under various comparison conditions. XGBoost classifiers showed the best classification performance among the three algorithms and all experimental conditions. To examine the influence of the features, we compared the feature importance results of XGBoost under all conditions. Differential asymmetry and frequency band power features were frequently found in high rank (i.e., top five importance). This trend can be verified in previous studies. Jenke et al. [57] suggested the utility of differential asymmetry in the emotion classification of EEG. In addition, Jatupaiboon et al. [58] and Li et al. [59] validated the influence of frequency band power values as features for emotion classification.

## 5. Conclusions

We collected and analyzed EEG signals in virtual environments to identify emotional states based on neural variations. To compare emotional influences in virtual content, we set four levels of emotional arousal (baseline, low, high arousal, and social anxiety) in our experimental materials. Three machine learning classification algorithms (XGBoost classifier, support vector classifier, and logistic regression) were applied to classify the neural fluctuations evoked by VR stimuli. Additionally, 54 features in five feature categories were applied to compare the usability for classification. Among the three classifiers, the XGBoost algorithm showed the best classification performance under all experimental conditions. Furthermore, we found the effectiveness of features, including differential asymmetry and frequency band power categories, to classify emotional states in our study design.

The first strength of this study was the application of machine learning classification algorithms to identify emotional neural states evoked by VR content. Second, the efficiency of the features was investigated through the feature importance of machine learning algorithms. Third, we collected EEG signals in VR content to examine the influence of the virtual environment on emotion. Finally, our framework can utilize an emotional state classification framework for various objectives. Our study has some limitations. First, we extracted and compared several features, including five feature categories. Other features or indices may be valuable for classification. However, we selected 54 features widely used in previous studies. Second, various methodologies, including deep learning algorithms, can be used in our research topics. In our study, we utilized machine learning models to check the importance of the features in the models. Third, additional validation is required in future studies with more participants to generalize our framework and results.

## Figures and Tables

**Table 1:** Extracted features from EEG signals.

Feature Category	Feature	No. of Features
Frequency band power (FP)	Delta power	20 features (4 electrodes × 5 band power)
	Theta power	
	Alpha power	
	Beta power	
	Gamma power	
Differential asymmetry (DASM)	Delta power	10 features (2 electrode pairs × 5 band power)
	Theta power	
	Alpha power	
	Beta power	
	Gamma power	
Rational asymmetry (RASM)	Delta power	10 features (2 electrode pairs × 5 band power)
	Theta power	
	Alpha power	
	Beta power	
	Gamma power	
Correlation coefficient (CC)	Delta power	10 features (2 electrode pairs × 5 band power)
	Theta power	
	Alpha power	
	Beta power	
	Gamma power	
Fractal dimension (FD)	AF7	4 features (4 electrodes)
	AF8	
	TP9	
	TP10	

**Table 2:** Coefficient values of each feature from lasso and ridge regression models.

Rank	Ridge Regression Coefficient	Feature	Lasso Regression Coefficient	Feature	No.	Selected Features (Common Feature)
1	1.6540	CC_Gamma_AF ^1^	1.6025	CC_Gamma_AF	1	CC_Gamma_AF
2	1.5410	CC_Gamma_TP	1.1770	CC_Gamma_TP	2	CC_Gamma_TP
3	1.4678	DASM_Beta_TP ^2^	0.7070	FP_Alpha_AF7	3	DASM_Beta_TP
4	1.2270	FP_Delta_TP9 ^3^	0.6682	DASM_Beta_TP	4	DASM_Delta_TP
5	1.2219	DASM_Delta_TP	0.5537	CC_Beta_AF	5	FP_Beta_TP9
6	1.2061	FP_Beta_TP9	0.5525	FP_Beta_TP9	6	FP_Alpha_AF7
7	1.1511	FP_Alpha_AF7	0.5366	FP_Beta_AF8	7	CC_Beta_AF
8	1.1342	RASM_Delta_AF ^4^	0.2972	CC_Delta_AF	8	FP_Beta_AF8
9	1.0362	CC_Beta_AF	0.2726	DASM_Alpha_AF	9	FP_Gamma_TP9
10	1.0280	DASM_Gamma_TP	0.2453	FP_Alpha_TP10	10	CC_Delta_AF
11	0.8683	FP_Beta_AF8	0.2225	FP_Gamma_AF8	11	DASM_Alpha_AF
12	0.8216	FP_Gamma_TP9	0.1509	DASM_Delta_TP	12	FP_Alpha_TP10
13	0.5619	RASM_Gamma_TP	0.1448	FP_Theta_TP9	13	DASM_Theta_AF
14	0.5369	CC_Delta_AF	0.1327	FP_Theta_AF7	14	FP_Theta_AF7
15	0.4626	DASM_Alpha_AF	0.1064	FP_Delta_AF8		
16	0.4328	RASM_Beta_TP	0.0798	DASM_Gamma_TP		
17	0.4295	RASM_Theta_AF	0.0751	FP_Delta_AF7		
18	0.3106	FP_Alpha_TP10	0.0561	DASM_Theta_AF		
19	0.2201	DASM_Theta_AF	0.0329	DASM_Beta_AF		
20	0.1270	FP_Theta_AF7	0.0081	FP_Gamma_TP9		

^1^ CC, correlation coefficient; ^2^ DASM, differential asymmetry; ^3^ FP, frequency band power; ^4^ RASM, rational asymmetry.

**Table 3:** Hyperparameters of three machine learning classifiers.

Classifier	Hyperparameter	Argument
XGBoost classifier	Eta	0.3
	Gamma	0
	max_depth	6
	min_child_weight	1
Support vector classifier	Kernel	rbf
	Gamma	auto
Logistic regression	Penalty	L2
	Solver	newton-cg

**Table 4:** Classification performance results for classifiers in binary-class condition.

Condition	Classifier	Precision	Recall	F1-Score	Accuracy	AUROC ^1^
Baseline vs. low arousal	XGBoost	0.846	0.846	0.838	0.849	0.925
	SVC ^2^	0.795	0.829	0.764	0.737	0.789
	LR ^3^	0.533	0.563	0.528	0.522	0.583
Baseline vs. high arousal	XGBoost	0.851	0.855	0.858	0.838	0.860
	SVC	0.769	0.747	0.748	0.722	0.686
	LR	0.651	0.673	0.663	0.632	0.669
Baseline vs. social anxiety	XGBoost	0.929	0.914	0.915	0.929	0.941
	SVC	0.843	0.833	0.860	0.830	0.856
	LR	0.721	0.728	0.733	0.712	0.813
low arousal vs. high arousal	XGBoost	0.853	0.858	0.880	0.843	0.858
	SVC	0.757	0.751	0.752	0.750	0.814
	LR	0.740	0.696	0.717	0.704	0.778
low arousal vs. social anxiety	XGBoost	0.865	0.840	0.852	0.840	0.857
	SVC	0.777	0.788	0.743	0.739	0.814
	LR	0.514	0.555	0.573	0.558	0.474
high arousal vs. social anxiety	XGBoost	0.903	0.921	0.907	0.892	0.936
	SVC	0.839	0.854	0.826	0.853	0.855
	LR	0.787	0.743	0.754	0.757	0.813

^1^ AUROC: area under the ROC curve, ^2^ SVC: support vector classifier, ^3^ LR: logistic regression.

**Table 5:** Classification performance results for classifiers in multi-class condition.

Condition	Classifier	Precision	Recall	F1-Score	Accuracy	AUROC ^1^
Baseline vs. low arousal vs. high arousal	XGBoost	0.912	0.911	0.913	0.938	0.938
	SVC ^2^	0.677	0.670	0.671	0.679	0.631
	LR ^3^	0.579	0.572	0.527	0.531	0.578
Baseline vs. low arousal vs. social anxiety	XGBoost	0.847	0.844	0.839	0.860	0.856
	SVC	0.707	0.758	0.754	0.745	0.767
	LR	0.537	0.532	0.564	0.547	0.534
low arousal vs. high arousal vs. social anxiety	XGBoost	0.902	0.911	0.911	0.938	0.905
	SVC	0.745	0.726	0.755	0.734	0.764
	LR	0.660	0.664	0.651	0.664	0.725
Baseline vs. low ^4^ vs. high arousal vs. social anxiety	XGBoost	0.843	0.874	0.846	0.845	0.858
	SVC	0.730	0.752	0.752	0.742	0.683
	LR	0.629	0.619	0.533	0.517	0.556

^1^ AUROC, area under the ROC curve; ^2^ SVC, support vector classifier; ^3^ LR, logistic regression; ^4^ low, low-arousal condition.

**Table 6:** Top 5 important features from XGBoost classifier in binary-class conditions.

Rank	Baseline vs. Low ^1^	Baseline vs. High ^2^	Baseline vs. Social ^3^	Low vs. High	Low vs. Social	High vs. Social
1	FP_beta_TP9	CC_delta_AF	DASM_delta_TP	DASM_delta_TP	FP_theta_AF7	DASM_delta_TP
2	DASM_alpha_AF	FP_alpha_TP10	FP_theta_AF7	CC_gamma_TP	DASM_delta_TP	FP_theta_AF7
3	CC_delta_AF	DASM_alpha_AF	DASM_theta_AF	FP_alpha_TP10	CC_gamma_TP	FP_beta_AF8
4	FP_alpha_AF7	CC_beta_AF	DASM_beta_TP	DASM_theta_AF	FP_beta_AF8	DASM_alpha_AF
5	DASM_beta_TP	FP_theta_AF7	FP_alpha_AF7	CC_beta_AF	CC_delta_AF	FP_beta_TP9

^1^ low: low-arousal condition, ^2^ high: high-arousal condition, ^3^ social anxiety condition.

**Table 7:** Top 5 important features from XGBoost classifier in multi-class conditions.

Rank	Baseline vs. Low ^1^ vs. High ^2^	Baseline vs. Low ^1^ vs. Social ^3^	Low vs. High vs. Social	Baseline vs. Low vs. High vs. Social
1	DASM_theta_AF	FP_theta_AF7	DASM_delta_TP	FP_theta_AF7
2	CC_delta_AF	DASM_alpha_AF	FP_theta_AF7	DASM_delta_TP
3	DASM_alpha_AF	FP_alpha_TP10	DASM_alpha_AF	CC_gamma_TP
4	DASM_delta_TP	FP_beta_AF8	CC_beta_AF	FP_alpha_TP10
5	DASM_beta_TP	FP_beta_TP9	DASM_theta_AF	CC_delta_AF

^1^ low: low-arousal condition, ^2^ high: high-arousal condition, ^3^ social anxiety condition.

## Data Availability

Not applicable.
